# Molecular Analysis of Bacterial Communities in Biofilms of a Drinking Water Clearwell

**DOI:** 10.1264/jsme2.ME12035

**Published:** 2012-10-10

**Authors:** Minglu Zhang, Wenjun Liu, Xuebiao Nie, Cuiping Li, Junnong Gu, Can Zhang

**Affiliations:** 1 School of Environment, Tsinghua University, Haidian District, Beijing, 100084, China; 2 Water Quality Monitoring Center, Beijing Waterworks Group, Chaoyang District, Beijing, 100085, China; 3 Institute of Hygiene and Environmental Medicine, Academy of Military Medical Sciences, Heping District, Tianjin, 30050, China

**Keywords:** drinking water clearwell, biofilm, T-RFLP, pyrosequencing, *Sphingomonas*

## Abstract

Microbial community structures in biofilms of a clearwell in a drinking water supply system in Beijing, China were examined by clone library, terminal restriction fragment length polymorphism (T-RFLP) and 454 pyrosequencing of the amplified 16S rRNA gene. Six biofilm samples (designated R1–R6) collected from six locations (upper and lower sites of the inlet, middle and outlet) of the clearwell revealed similar bacterial patterns by T-RFLP analysis. With respect to the dominant groups, the phylotypes detected by clone library and T-RFLP generally matched each other. A total of 9,543 reads were obtained from samples located at the lower inlet and the lower outlet sites by pyrosequencing. The bacterial diversity of the two samples was compared at phylum and genus levels. Alphaproteobacteria dominated the communities in both samples and the genus of *Sphingomonas* constituted 75.1%–99.6% of this phylum. A high level of *Sphingomonas* sp. was first observed in the drinking water biofilms with 0.6–1.0 mg L^−1^ of chlorine residual. Disinfectant-resistant microorganisms deserve special attention in drinking water management.

This study provides novel insights into the microbial populations in drinking water systems and highlights the important role of *Sphingomonas* species in biofilm formation.

Since the first description of microbial biofilms by Zobell in 1943 (37), they remain a great concern in a wide range of fields, including drinking water systems. Biofilms play an important role in the safety of drinking water, because bacteria in biofilms are more resistant to disinfection than those in bulk water (17). A biofilm is a product of microorganisms aggregated on a surface, which is composed primarily of bacteria, protein and extracellular polymeric substances (EPS) generated by the microorganisms (8). Biofilms in drinking water systems are responsible for the loss of disinfectant residuals in water distribution pipelines, increased potential for the survival of pathogens, reduction of dissolved oxygen, taste and odor changes, red or black water problems, clogging and corrosion, and reduced life expectancy of pipeline materials (11, 13, 22, 25, 26, 31).

A large number of microorganisms, e.g. *Pseudomonas*, *Legionella*, *Methylophilus*, *Bacillus*, *Arthrobacter*, *Acinetobacter*, and *Flavobacterium* have been identified to form biofilms and grow in the submerged surface of water treatment plants and distribution pipelines (10, 18, 32). Biofilms in drinking water systems might also serve as environmental reservoirs for pathogenic microorganisms, because the abundance of biofilm-forming bacteria (including *Aeromonas*, *Legionella*, *Pseudomonas*, *Salmonella*, and *Vibrio*) may cause waterborne diseases (23, 30). However, most studies related to drinking water biofilms focused either on laboratory model systems or distribution systems, which may not provide a true representation of the in situ biofilm problem at the endpoint of the waterworks. To date, no studies have demonstrated the bacterial community structures in drinking water clearwell biofilms. A clearwell stores treated drinking water before it is sent throughout the city to consumers. In order to ensure adequate disinfection, treated water must be in contact with disinfectant to effectively kill bacteria, viruses, and parasites that might have been present in the raw water; however, bacteria may enter the bulk water by release from the biofilm or by the sloughing of biofilm particles from hydraulic shearing. In addition, biofilms have been shown to mitigate the effectiveness of commonly used disinfectants. As a result, the formation of biofilm increases the survival of bacteria and their regrowth in the distribution systems. The need to understand basic information about bacterial community in clearwell biofilms has a high priority.

In most previous studies, the composition and dynamics of the microbial communities in drinking water biofilms were shown through cultivation and traditional molecular methods, such as the 16S rDNA clone library, denaturing gradient gel electrophoresis (DGGE) and fluorescent in situ hybridization (FISH) (19, 24, 29); however, biofilms contain a vast majority of microorganisms that have not yet been cultured or cannot be detected by traditional molecular tools. Detailed information about drinking water biofilms is still lacking. Next-generation DNA sequencing, tag pyrosequencing technology, provides a new method for in depth analysis of microbial diversity. This method allows the detection of rare microbial populations in environmental samples. Thus, it is a promising tool for microbial ecology analysis of drinking water systems with a low bacterial level; however, this technique has not yet been fully utilized to study the bacterial communities of drinking water biofilms. To the best of our knowledge, only two published studies have employed pyrosequencing to investigate bacterial biofilms in drinking water systems (10, 15).

In this study, the microbial composition of biofilm samples collected from a clearwell in a full-scale drinking water plant in Beijing was investigated by 16S rDNA based clone library, T-RFLP fingerprinting and 454 pyrosequencing. This study contributes to the identification and understanding of the bacterial community structure that colonizes natural drinking water biofilms and lays the foundation for developing water treatment and disinfection strategies to combat biofilm formation in drinking water production.

## Materials and Methods

### Sample collection

Biofilm samples used in this study were collected from the walls of a water supply clearwell in a drinking water treatment plant located in Beijing. It currently produces up to 1.5 million cubic meters (39.6 million gallons) per day and meets about 60% of the city’s drinking water demands. This facility was built in the 1980s to treat surface water from water reservoirs on the outskirts of Beijing. The treatment procedure consists of the following five processes: coagulation, sedimentation, filtration, activated carbon adsorption and chlorine disinfection. Finished water is pumped out to endpoint customers from the clearwell. The clearwell is built of concrete and steel, and its dimensions are 195 m long and 120 m wide, with a maximum available depth of 4.7 m. The dead storage level is 0.5 m deep. At the time of this study, this clearwell had been in operation for five years since the last time it had been cleaned.

For bacterial analysis, biofilms on the walls of the clearwell were scraped off with sterile cotton swabs and transferred into sterile tubes. Each sample was obtained from an area of approximately 5 cm×5 cm. Six biofilm samples were obtained from the inlet, middle and outlet sections of the clearwell, in lower and upper regions (about 0.5 m and 2.5 m from the bottom) of each section, respectively (designated R1–R6). The normal total chlorine concentration of inlet is 1–2 mg L^−1^ and the chlorine residual is about 0.6–1.0 mg L^−1^ at the outlet of the tank. The hydraulic retention time (HRT) is 3–5 hrs. All samples were transported to the laboratory within 1 h and stored at −20°C until further analysis.

### DNA extraction and 16S rRNA gene PCR amplification

The cotton swabs were suspended in phosphate-buffered saline (PBS, pH 7.0) and biofilms were eluted by vigorous vortexing. The suspensions were centrifuged to collect the pellet. Genomic DNA was extracted from six biofilm samples using the MoBio ultraclean soil DNA kit (MoBio Laboratories, Carlsbad, CA, USA) according to the manufacturer’s instructions. 16S rRNA genes were amplified using the universal eubacterial primers, 27F (5′-AGAGTTTGATC MTGGCTCAG-3′) and 1492R (5′-GGTTACCTTGTTACGACTT-3′) (16). The PCR mixture contained 1×PCR buffer (Promega, Madison, WI, USA), 2.5 mM MgCl_2_, 200 μM concentrations of each deoxynucleoside triphosphate, 400 nM of each forward and reverse primer, 1 U Taq polymerase (Promega) and 1 μL DNA template in a total of 25 μL reaction mixture. The PCR reactions were run on an Eppendorf Mastercycler (Eppendorf, Germany) with the following thermal profile: 2 min initial denaturation at 94°C followed by 30 cycles at 94°C for 1 min, 55°C for 1 min, and 72°C for 1 min, with a final extension at 72°C for 10 min and a hold at 4°C. PCR products were purified using PCR purification Kit (Biomiga, USA). A clone library was developed using R1 as the template. The purified PCR fragments were ligated into a pEASY-T cloning vector and cloned into Trans1 chemically competent cells according to the manufacturer’s instructions (TransGene, China). Transformants were screened by blue/white colony selection on agar containing X-gal/IPTG and 100 μg mL^−1^ ampicillin. White colonies were randomly selected and grown overnight in 3 mL LB medium containing 100 μg mL^−1^ ampicillin. Plasmids were isolated using a plasmid purification kit (Biomiga). The insert in the plasmid was checked by PCR using primers M13F and M13R as previously described (4). Twenty-two positive clones were selected and submitted for 16S rDNA sequencing. Clone library coverage was calculated by Good’s coverage (1-n/N)×100, where n is the number of single reads and N is the number of total reads.

### T-RFLP analysis

The bacterial 16S rRNA gene was amplified using eubacterial universal primers 8F (5′-AGAGTTTGATCCTTGGCTCAG-3′) and 1492R. The forward primer 8F was labeled at the 5′ end with 6-FAM (carboxyfluorescein). The fluorescent PCR products were cleaned using a PCR purification kit (Biomiga). A total of 10 μL purified product was digested with 3U MspI restriction enzyme (Promega, USA) for 4 h at 37°C, followed by an inactivation step at 65°C for 10 min. The final reactions were submitted to Sunbioech (Beijing, China) for sequencing using ABI prism 3100 capillary sequencing.

T-RFLP profiles were analyzed using Peak Scanner software (Applied Biosystems/Life Technologies, Carlsbad, CA, USA) to determine the richness and relative abundance of each T-RF (terminal restriction fragment) in a sample. Fragments peaks under 50 fluorescent units and smaller than 50 bp or larger than 500 bp were excluded from further analysis. T-RFs that differed by less than 1 bp were clustered. The relative abundance of a given T-RF was calculated by dividing the area of each peak by the cumulative peak area of that sample. T-RF richness was calculated as the number of peaks in each sample profile.

To identify the dominant peaks in T-RFLP profiles, the 22 positive clones were submitted for T-RFLP analysis. T-RF peaks of each individual clone were compared with the T-RFLP profiles of the biofilm samples. The species that dominant peaks represented were identified based on the results of 16S rRNA gene sequencing and the T-RFLP profiles of clones.

### 454 pyrosequencing and data analysis

The V1–V3 region of the 16S rRNA genes was amplified with universal bacterial primers Gray 28F (5′-GAGTTTGATCNTGGCT CAG-3′) and Gray 519R (5′-GTNTTACNGCGGCKGCTG-3′) (9). Tag-encoded FLX amplicon pyrosequencing analyses were performed on a Roche 454 FLX instrument at the Research and Testing Laboratory (RTL) based upon RTL protocols (Lubbock, TX, USA).

Following sequencing, all failed sequence reads, low quality sequence ends and tags and primers were removed. Sequences were processed using the QIIME software package (3). Raw sequence reads were excluded if they were shorter than 200 bases in length, had a mean quality score less than 25, or did not contain a primer and barcode sequence. Similar sequences were clustered into Operational Taxonomic Units (OTUs) using a minimum identity of 97% by UCLUST software (6). Taxonomy was assigned to each unique sequence using the Ribosomal Database Project (RDP) classifier with a minimum support threshold of 80% and the RDP taxonomic nomenclature. Good’s coverage percentage was calculated as described above. Equitability, phylogenetic diversity (PD), abundance-based coverage estimator (ACE), Chao1 richness estimator, Shannon and Simpson diversity indices were calculated by QIIME pipeline (3). Pearson correlation were calculated by Mothur to evaluate statistical differences between two samples (28).

Raw sequences were deposited in the National Center for Biotechnology Information, utilizing the Sequences Read Archive (SRA) with accession number SRA040071.11. Sequences of the clones were deposited in Genbank under accession numbers JQ029057–JQ029078.

## Results

### Bacterial community structures of biofilms revealed by T-RFLP and clone library

**Fig. 1:**
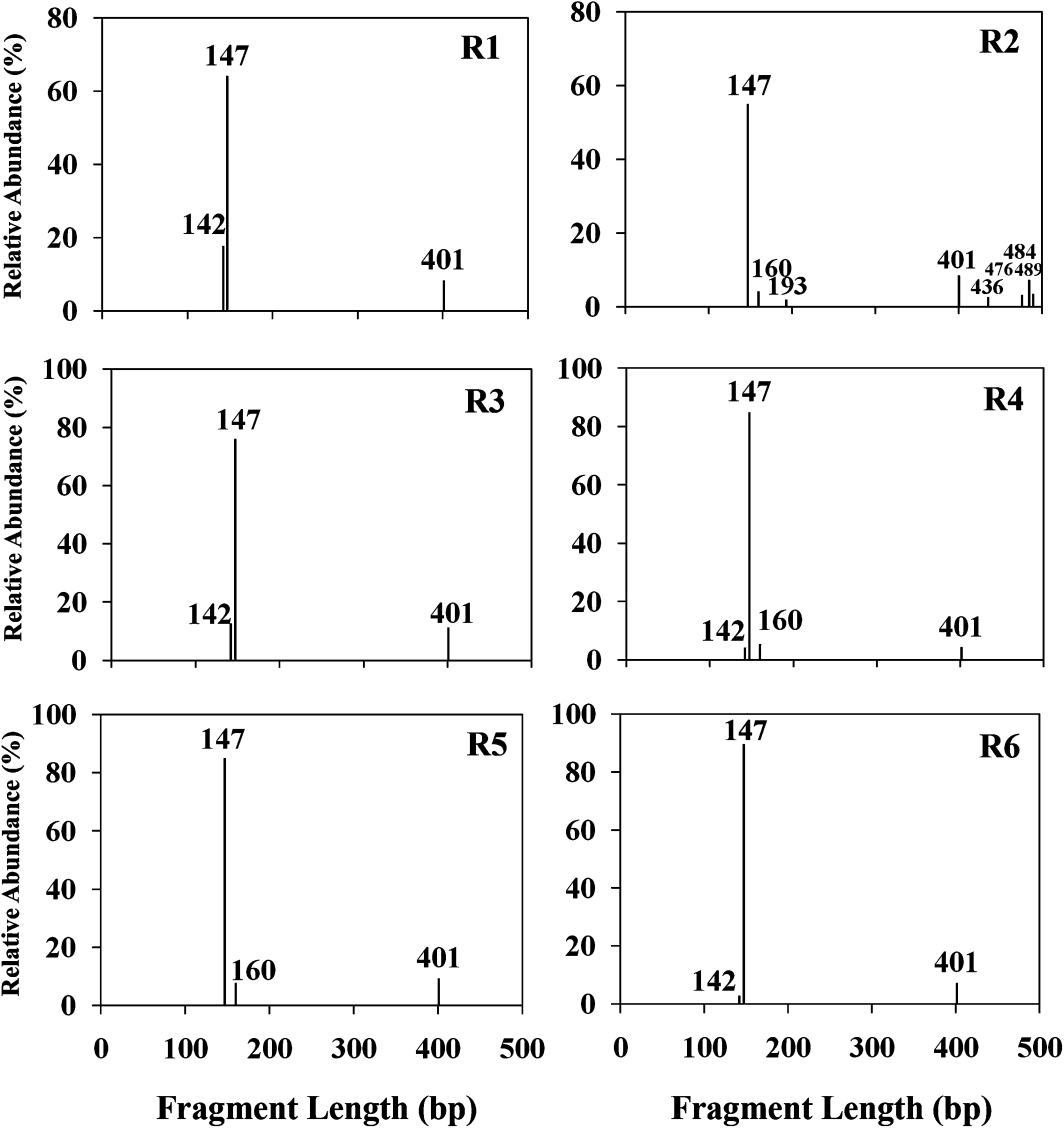
T-RFLP fingerprints of six biofilm samples from upper and lower sites of the inlet (R1 and R2), middle (R3 and R4) and outlet (R5 and R6) of the clearwell. 16S rDNA-based T-RFLP profiles were produced by digestion with *Msp*I. The fragment length of each T-RF in base pair size (bp) is given.

To compare the bacterial community profiles in different regions of the drinking water supply reservoir, six biofilm samples (R1–R6) collected at the entrance, middle and exit areas (lower and upper, respectively) were investigated by T-RFLP analysis (Fig. 1). T-RFLP fingerprinting of the six biofilm samples revealed similar patterns of bacterial diversity. Three to eight T-RFs from each sample were observed by MspI digestion. The mean bacterial richness of the three samples from the bottom (R2, R4 and R6) was slightly higher than samples from the top of the storage tank (R1, R3 and R5), although this was not statistically significant (*P*=0.057 by *t* test). The most dominant bacteria among all samples at 147 bp position were identified as *Sphingomonas* by 16S rDNA cloning. Twenty-two colonies were sequenened from one of the biofilm samples collected from the upper inlet (R1) and their phyogentic affiliation was determined using 16S rRNA gene analysis. Coverage of the clone library was 86%. Four OTUs obtained from the 22 clones belonged to *Sphingomonas* sp. *Chryseobacterium* sp. *Acinetobacter* sp. and *Rhodocyclus* sp. (Table S1). According to the BLAST results, the most dominant OTU related to *Sphingomonas*, which is concordant with T-RFLP analysis.

### Pyroequencing results

To further understand the bacterial community composition, two biofilm samples R2 and R6, located at the entrance and exit regions of the water tank, respectively, were selected for 454 pyrosequencing. A total of 21,093 raw sequences were generated from two samples. After trimming, sorting and quality control, 9,543 reads with an average read length of 365 bp were used for further analysis. The read length distribution of two samples is summarized in Fig. S1. At a cut-off of 97% sequence similarity, 155 bacterial OTUs were obtained, with 131 OTUs from R2 and 70 OTUs from R6, respectively. Forty-six OTU overlap was found between the two samples. To evaluate bacterial richness between the two biofilm samples, rarefaction curves of observed OTUs were plotted (Fig. S2). The curve of R6 started to flatten after 2,000 reads and Good’s coverage of R2 was 98.9%, indicating that the communities were well covered by the sequencing effort. The statistical estimates of species richness and diversity indices (Chao1, ACE diversity, Simpson, Shannon, PD and Equitability) in R2 were higher than in R6 (Table 1). The bacterial communities of the two samples revealed no statistically significant differences based on Pearson correlation (r=0.06, *P*=0.46).

### Bacterial community analysis at the phylum level

**Fig. 2:**
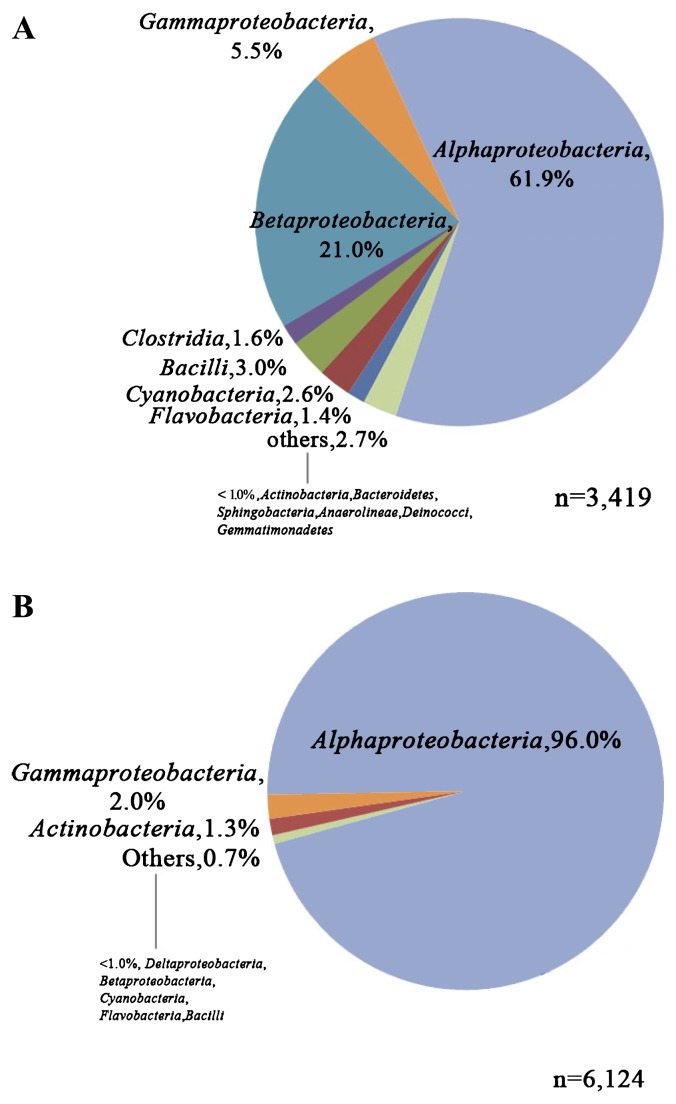
Relative abundance of phylogenetic groups in the biofilm samples determined at 97% similarity by RDP classifier. (A) R2: lower site of the inlet, (B) R6: lower site of the outlet. The names of bacteria in small characters represented the contents of others.

The phylogenetic classification of 16S rDNA sequences from the two samples is summarized in Fig. 2. The pyrosequencing results were in good agreement with those revealed by T-RFLP analysis, whereas a higher level of bacterial diversity was found in the inlet biofilm sample of R2. The most prominent population in both samples was Alphaproteobacteria, which was present at a relative abundance of 61.8% in sample R2 and 96.0% in sample R6, respectively (Fig. 2A and 2B). The second dominant group in R2, affiliated with Betaproteobacteria (21.0%), showed a much lower proportion in R6 (<1%). Gammaproteobacteria (5.5%) was the third abundant population in R2 and other genera in both samples were less than 3.0%. Except for Alphaproteobacteria and Actinobacteria, other bacterial phyla showed lower relative abundance values in R6 than the values in R2.

### Diversity and abundance of bacteria at the genus level

Further evaluation of taxonomic groups at the genus level was carried out to assess the bacterial diversity in two biofilm samples. Thirty-three bacterial genera identified in two samples are shown in Table 2. Six genera of *Streptococcus*, *Bacillaceae*, *Flavobacterium*, *Raoultella*, *Propionivibrio* and *Polyangiaceae* were only observed in sample R6, and the other 21 bacterial genera were only present in R2. The most dominant microorganisms belonged to genus *Sphingomonas*, representing 46.4% (R2) and 95.6% (R6) of the total sequences in each sample, respectively. Except for *Sphingomonas* sp., two other dominant populations, *Acidovorax (*11.6%) and *Sphingopyxis* (11.2%), were observed in sample R2. The relative abundance of all the other genera detected in the two samples was less than 5%. Genera that include important potential pathogens were also observed in two samples, such as *Acinetobacter*, *Clostridiaceae*, *Burkholderia*, *Pseudomonas* and *Brevundimonas*.

## Discussion

Three molecular analysis techniques, clone library, T-RFLP fingerprinting and pyrosequencing, were used to evaluate the bacterial composition of drinking water clearwell biofilms. Clone library and T-RFLP generally matched each other with respect to dominant populations of biofilm samples and our results agreed with a previous study that T-RFLP cannot show minor bacterial populations (20). Although a relative low number of clones were sequenced, 86% were assigned to the same genus, indicating the limitation of the clone library for the detection of minor species. Pyrosequencing has been reported to successfully reveal rare microbial groups that might have otherwise been undetected by other molecular techniques (21, 27). It provides high-depth analysis of the community structure and diversity of eubacteria in drinking water biofilms, which revealed more diverse bacterial communities than T-RFLP and the clone library. Compared with the clone library, T-RFLP is a quick, low-cost and high throughput tool to detect dominant bacterial groups in environmental samples and pyrosequencing can compensate for the disadvantage of T-RFLP fingerprinting in detecting minor populations in drinking water systems.

It is not surprising that the phylogenic diversity of biofilms determined by pyrosequencing was higher than in studies employing traditional molecular techniques (23, 36). The species richness of drinking water clearwell biofilms estimated by OTU numbers (70 and 113 OTUs) is much lower than that in a previous study based on pyrosequencing. Kwon et al. observed 1,133 OTUs in a membrane biofilm from a pilot-scale drinking water treatment plant with residual chlorine (15 mg L^−1^ NaOCl, equal to 14.3 mg L^−1^ total chlorine) supplied during the cleaning period (15). Alphaproteobacteria were predominant in the biofilm (32.8%), which is consistent with our results, whereas the OTU number identified in the study is similar to another previous report, which found that the OTUs of biofilms in two water meters were 133 and 208, respectively. The dominant bacterial groups in both water meters were Betaproteobacteria (52.9% and 71.3%, respectively). The chlorine residual in water meters (~3 mg L^−1^) was higher than that in the clearwell (0.6–2 mg L^−1^) (10). Therefore, the discrepancies of bacterial richness and diversity might not only be caused by the concentration of disinfectants, but also by the raw water quality, temperature, material of the surface where biofilms grew, and bacteria regrowth.

The detection of an extremely high level of *Sphingomonas* sp. is quite surprising. This OTU was classified into *Sphingomonas* and *Sphingobium* with 99% similarity based on the GenBank and RDP databases, respectively. The names of these two genera are disputed among bacteriologists. According to Takeuchi *et al.* (33), *Sphingobium* was proposed as a new genus in addition to *Sphingomonas*; however, Yabuuchi *et al.* (35) considered that *Sphingobium* should be placed in the genus of *Sphingomonas*. According to Rule 37a, bacteriologists adhering to this proposal may no longer use the genus name *Sphingobium*. Thus, this OTU was assigned as *Sphingomonas* both in clone library and 454 pyrosequencing in this study.

Although *Sphingomonas* sp. have been frequently identified from drinking water systems over the past few years (2, 10, 14, 34), the high abundance of *Sphingomonas* in biofilms has not been reported previously. Hong *et al.* found that *Sphingomonas* sp. accounted for 15% of the total bacterial population in the biofilms of a drinking water meters (10). *Sphingomonas* spp. have been shown to secrete expolysaccharides, which are the major component of microbial biofilms (12). Bereschenko *et al.* demonstrated that *Sphingomonads* were the key biofouling organisms on RO membranes and feed-side spacer surfaces. *Sphingomonads* can rapidly colonize the entire membrane and spacer surfaces and cover them with their EPS. *Sphingomonads* were also likely responsible for the initial biofilm formation (1). Our findings, together with the evidence of previous studies, indicated that *Sphingomonas* species play a crucial role in drinking water biofilm formation. Moreover, some *Sphingomonas* species have been recognized to play a role in human diseases, primarily by causing a range of mostly nosocomial and non-life-threatening infections (5); however, many sequences could not be assigned to species level based on the current database. Thus, 16S rDNA sequencing results may not able to identify pathogenic bacteria accurately.

In addition, *Sphingomonas* sp. identified in this clearwell with a high level of chlorine residual was presumed to exhibit a high chlorine-resistant property, as the relative abundance of *Sphingomonas* increased significantly from the entrance to the exit of the tank. Our results supported a previous study, which demonstrated that *Sphingomonas* was slightly tolerant to chlorine (7). In our previous study, a *Sphingomonas* strain, isolated from a model drinking water distribution system, was highly resistant to chlorine. Only a 5.26% inactivation rate was observed when the strain was treated with 4 mg L^−1^ chlorine for 60 min; however, this isolate was highly sensitive to UV irradiation, indicating that UV disinfection could be an alternative method for the control of *Sphingomonas* (unpublished results). In the next phase, the authors hope to isolate abundant bacteria belonging to *Sphingomonas* sp. to investigate the disinfection efficiency of chlorine/chloramines and UV irradiation and to find a strategy to control the growth of *Sphingomonas* sp. in drinking water systems. Moreover, bacterial community analysis of water samples from the source to the endpoint of the waterworks is needed to trace their origins.

Our study contributes to the systematic investigation of the composition and structure of bacterial communities in drinking water clearwell biofilms. The presence of chlorine-resistant microorganisms that may contain opportunistic pathogenic bacteria in the drinking water supply clearwell is an issue of great concern that deserves our special attention. Additional efforts to characterize the inactivation rate of *Sphingomonas* sp. by different disinfection strategies are needed and will be useful for improving our understanding of chlorine-resistant microorganisms.

## Supplementary Material



## Figures and Tables

**Table 1:** Comparison of coverage and diversity indices from pyrosequencing analysis

Sample ID	Reads	OTUs	Good’s coverage	Equitability	Chao1	ACE	Shannon	Simpson	PD
R2	3,419	131	98.9	0.57	160	178	4.02	0.810	6.90
R6	6,124	70	99.8	0.22	78	79.5	1.33	0.270	4.03

OTUs were defined with 97% similarity, Equitability, richness estimators (ACE and Chao1), and diversity indices (PD, Shannon and Simpson) were calculated using QIIME pipeline.

**Table 2:** Relative abundance of bacterial genera in two biofilm samples. 16S rDNA sequences obtained from pyrosequencing were classified by RDP classifier at 97% similarity

Relative abundance (%)	R2	R6

Genera		
*Sulfurimonas*	0.03	—
*Enhydrobacter*	0.03	—
*Moraxella*	0.03	—
*Shigella*	0.06	—
*Pseudomonas*	0.06	0.18
*Polaromonas*	0.06	—
*Xiphinematobac*	0.09	—
*Micrococcineae*	0.09	—
*Chryseobacterium*	0.12	—
*Bosea*	0.29	—
*Corynebacterineae*	0.32	1.34
*Gemmatimonas*	0.32	—
*Rhodobacter*	0.38	0.05
*Burkholderia*	0.53	—
*Bacteroides*	0.56	—
*Deinococcus*	0.97	—
*Stenotrophomonas*	1.08	1.03
*Wolbachia*	1.23	—
*Brevundimonas*	1.52	—
*Clostridiaceae*	1.64	—
*Lactobacillus*	3.01	—
*Acinetobacter*	3.19	—
*Comamonadaceae*	3.57	—
*Shinella*	4.18	—
*Sphingopyxis*	11.17	0.18
*Acidovorax*	11.64	—
*Sphingomonas*	46.45	95.57
*Bacillaceae*	—	0.02
*Raoultella*	—	0.03
*Propionivibrio*	—	0.05
*Polyangiaceae*	—	0.05
*Flavobacterium*	—	0.13
*Streptococcus*	—	0.33
