# Supplementary material for: Molecular Analysis of Bacterial Communities in Biofilms of a Drinking Water Clearwell
Source: Microbes Environ. 2012 Oct 10;27(4):443–8. doi: 10.1264/jsme2.ME12035 (PMC4103552; doi:10.1264/jsme2.ME12035)
Supplement: Supplementary file 1 [file 27_443_s1.pdf]

Supporting Information for

Molecular analysis of bacterial communities in biofilms of a drinking water clearwell

Minglu Zhang[1], Wenjun Liu[1*], Xuebiao Nie[1], Cuiping Li[1], Junnong Gu[2], Can Zhang[1]

1. School of Environment, Tsinghua University, Beijing, China, 100084

2. Water Quality Monitoring Center, Beijing Waterworks Group, Beijing, China,100085

[*] Corresponding author. E-mail: wjliu@tsinghua.edu.cn;
Tel:+86-10-62782196; Fax: +86-10-62797643.

Table S1. The closest matched strains of the OTUs according to GenBank and RDP databases based on 16S rDNA clone library.

OTU	No. of Clones	Percentage	Closest strains in *GenBank* database	Closest strains in RDP database
1	19	86.4%	*Sphingomonas* sp. (99%)	*Sphingobium* (99%)
2	1	4.5%	*Chryseobacterium* sp. (99%)	*Chryseobacterium* (71%)
3	1	4.5%	*Acinetobacter* sp. (100%)	*Acinetobacter* (100%)
4	1	4.5%	*Rhodocyclus* sp. (99%)	*Propionivibrio* (78%)

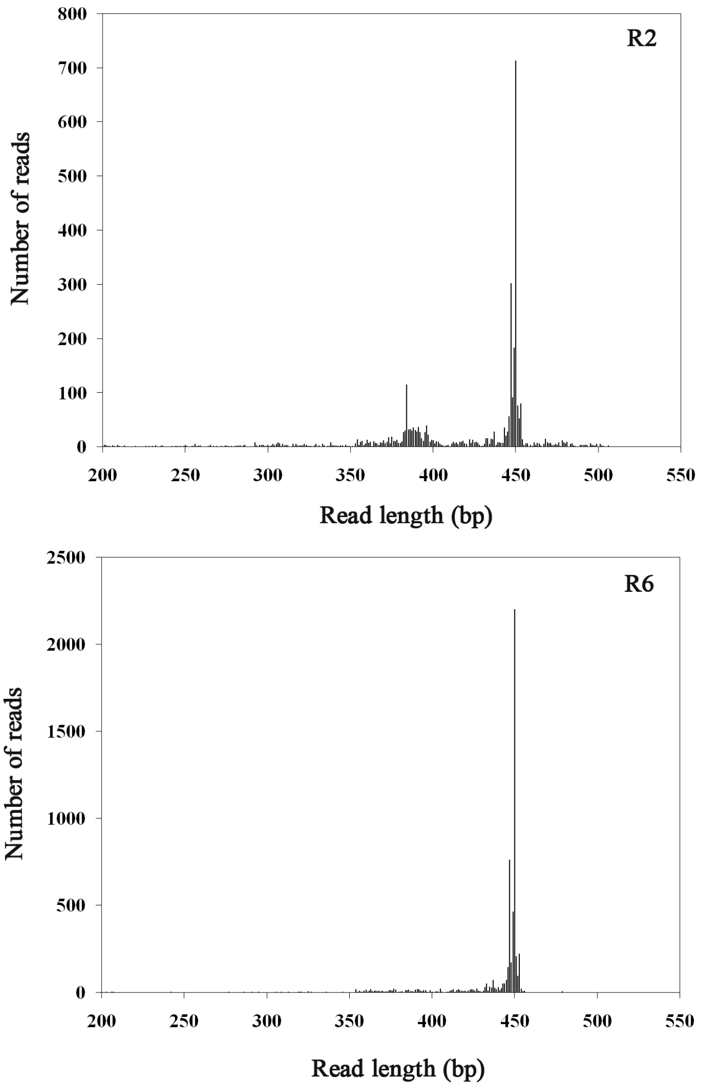

Fig. S1. Histogram of trimmed read length from two biofilm samples based on pyrosequencing.

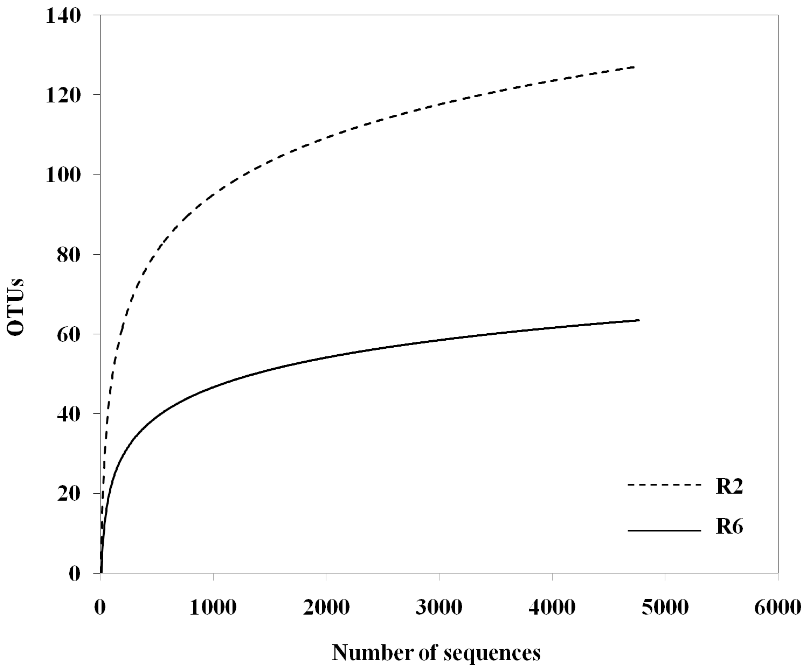

Fig. S2. Rarefaction analysis of the two biofilm samples determined at 97% similarity. R2: lower site of the inlet, R6: lower site of the outlet.
